# Predicting Human Genetic Interactions from Cancer Genome Evolution

**DOI:** 10.1371/journal.pone.0125795

**Published:** 2015-05-01

**Authors:** Xiaowen Lu, Wout Megchelenbrink, Richard A. Notebaart, Martijn A. Huynen

**Affiliations:** 1 Department of Bioinformatics, Radboud Institute for Molecular Life Sciences, Radboud University Medical Centre, Nijmegen, The Netherlands; 2 Institute for Computing and Information Sciences, Radboud University Nijmegen, Nijmegen, The Netherlands; 3 Centre for Systems Biology and Bioenergetics, Radboud University Medical Centre, Nijmegen, The Netherlands; Johns Hopkins University, UNITED STATES

## Abstract

Synthetic Lethal (SL) genetic interactions play a key role in various types of biological research, ranging from understanding genotype-phenotype relationships to identifying drug-targets against cancer. Despite recent advances in empirical measuring SL interactions in human cells, the human genetic interaction map is far from complete. Here, we present a novel approach to predict this map by exploiting patterns in cancer genome evolution. First, we show that empirically determined SL interactions are reflected in various gene presence, absence, and duplication patterns in hundreds of cancer genomes. The most evident pattern that we discovered is that when one member of an SL interaction gene pair is lost, the other gene tends not to be lost, i.e. the absence of co-loss. This observation is in line with expectation, because the loss of an SL interacting pair will be lethal to the cancer cell. SL interactions are also reflected in gene expression profiles, such as an under representation of cases where the genes in an SL pair are both under expressed, and an over representation of cases where one gene of an SL pair is under expressed, while the other one is over expressed. We integrated the various previously unknown cancer genome patterns and the gene expression patterns into a computational model to identify SL pairs. This simple, genome-wide model achieves a high prediction power (AUC = 0.75) for known genetic interactions. It allows us to present for the first time a comprehensive genome-wide list of SL interactions with a high estimated prediction precision, covering up to 591,000 gene pairs. This unique list can potentially be used in various application areas ranging from biotechnology to medical genetics.

## Introduction

A synthetic lethal (SL) genetic interaction is defined as a functional relationship between two genes where the loss of either gene is viable but the loss of both is lethal [1]. A comprehensive map of SL interactions sheds light on the relationships between genotype and phenotype[2–5], potentially advancing the understanding of the mechanisms of complex human disease[6, 7], and even providing therapeutic treatment strategies for human diseases such as cancer[8]. For instance, several studies have shown that inhibiting one gene in an SL pair could be lethal to cancer cells in which the other gene of that pair is mutated [9–11]. The underlying concept is that, in a cancer cell, a mutation in one (A) of the two genes in an SL pair (A-B), which is not mutated in the normal cell, allows for selectively killing tumor cells by inhibiting B. Despite recent breakthroughs in technologies to identify SL interactions on a genome-wide scale [12–15], these interactions remain largely unknown in human, underlining the need for predictive computational approaches.

**Fig 1:**
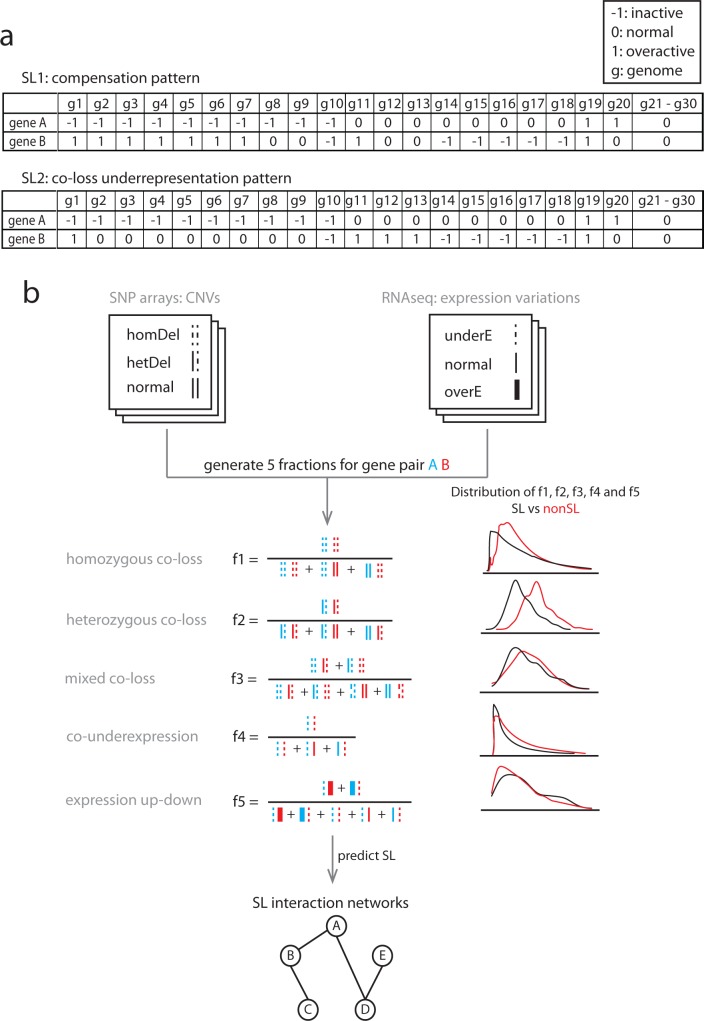
Patterns across cancer genomes reflecting selection against gene co-inactivation, and the workflow to predict SL interactions. **(a)** A SL interaction SL1 between gene A and B can show a ‘compensation’ pattern across cancer genomes in which it is more likely that when A is inactive (denoted by -1), B is overactive (denoted by 1) to compensate the inactive A (genomes 1–10), compared to when A is active (genomes 11–30). SL interaction SL2 can, show a ‘co-loss underrepresentation’ in which a combined loss of A and B (denoted by -1 and -1, genome 10) across cancer genomes is underrepresented compared to a loss of either one of the two (genomes 2–9 and genome 14–18). Note that SL1 can also be identified via the co-loss underrepresentation pattern, but the SL2 can only be identified via the co-loss underrepresentation pattern. **(b)** The model requires two types of data as input, i) CNVs measured by SNP arrays and ii) gene expression variations measured by RNAseq. In CNVs, the status of a gene can be a homozygous deletion (two dash lines), a heterozygous deletion (one dash and one solid line) or normal (two solid lines). For CNVs, we generated three fractions to quantify the likelihood that a gene pair has a homozygous co-loss (f1), a heterozygous co-loss (f2) or a mixed co-loss (f3) event. In gene expression variations, a gene can be under-expressed (one dash line), normal (one solid line) or over-expressed (one bold line). For expression status, we generated two fractions, f4 and f5. f4 is the likelihood that both genes in a gene pair are under-expressed. f5 is the likelihood that a gene pair has an expression up-down event where one is over-expressed while the other one is under-expressed. All these five fractions showed a distribution difference between SL and non-SL pairs. By integrating these five fractions into a prediction model, we can identify SL interactions that can be presented as a network.

Previous computational approaches have mostly been developed to predict SL interactions in model microorganisms, such as *Saccharomyces cerevisiae* and *Caenorhabditis elegans*[16–18]. However, genetic interactions are not strongly conserved between species, for instance only 29% of genetic interactions were found to be conserved between the fungi *S*.*cerevisiae* and *Schizosaccharomyces pombe*[19] and the conservation of SL interactions between microorganisms and human still has to be established. Recently, a study proposed to use cancer genomic data [20] to identify SL interactions by using a ‘compensation‘ pattern: one gene (A) is inactive while the other one (B) is highly active, thereby selecting against the situation that both genes become lost and, as such, causing a lethal phenotype. We recently showed another genomic pattern of SL interacting gene pairs: SL interactions are reflected in present-day species genomes and their ancestral genomes in a way that the combined loss of two genes in an SL pair does not frequently occur across evolutionary history [21]. This raises the question whether we can use this ‘co-loss underrepresentation’ pattern to predict SL pairs from human cancer genomes (Fig 1A). Here, we used copy number variations, i.e. gene loss or gene gain, across hundreds of cancer genomes to ask i) are empirical SL interactions reflected in cancer genome evolution and, if so, ii) which gain and loss patterns correlate most with SL interactions, and iii) can they be captured into a simple computational model to predict SL interactions genome widely?

By exploiting the availability of gene expression data for a large number of cancer samples[22] and recent empirically measured SL interactions in human[23, 24], we found that genes with SL interactions are more likely to have an expression pattern where one gene is over-expressed while the other one is under-expressed, thereby confirming earlier observations[20]. Strikingly we observed that SL pairs are less likely to be co-lost and co-under expressed than non-SL gene pairs. On the basis of these findings, we present a simple ensemble-based computational model that captures the genomic patterns to predict genome-wide SL pairs with high accuracy. We provide a unique and comprehensive map of the human SL interaction network with a high estimated prediction precision of 67%, i.e., 14-fold higher than expected from chance, covering 591,000 pairs. This map is expected to be highly valuable in the light of understanding human disease and designing therapeutic strategies.

## Materials and Methods

### Data sources

We retrieved the experimentally measured SL pairs and non-SL pairs from two studies[23, 24]. We collected 297 SL pairs and 6358 non-SL pairs in total. After excluding the pairs of which both genes are located on the same chromosome, we obtained 270 SL pairs and 5660 non-SL pairs (S1 Table).

The CNV data is directly retrieved from the cBioPortal for Cancer Genomics[25]. The CNV signals in the database are generated as homozygous deletion, heterozygous deletion, normal copy, duplication and amplification. Using the ‘cgdsr’ R-package, we obtained the CNV data for 14136 tumor patients from 31 cancer types.

The RNAseq data are obtained from the Broad Institute’s Genome Data Analysis Center (GDAC) Firehose[26]. The link for downloading the RNAseq data is http://gdac.broadinstitute.org/runs/stddata__2014_03_16/data. For each cancer study, we first downloaded the files named as ‘_RSEM_genes_normalized_data.Level_3’, which contains the estimated expression levels for each gene in human genome from RNAseq data by using the RSEM package[27]. In total we collected an expression profile for 7362 tumor patients with coverage of 26 cancer types. Then, for each gene in a tumor, we computed the Z-score and P-value to infer its over- or under-expression relative to expression levels in normal tissue. If at least 25 normal samples from the same tissue type as that of the cancer are available, we used this as the comparison set. Otherwise, all normal tissue samples, regardless of the tissue specificity, were used. The numbers of normal samples for each type of tumor are listed in S2 Table. To adjust for multiple hypothesis testing, we used the False Discovery Rate (Benjamini-Hochberg) method to adjust p-values[28, 29] in R. A cutoff of the adjusted P-value, 0.05, was applied to generate the over- or under-expression signal.

### Extract the pattern for SL pairs from genomic variations

The copy number variations can be, -2 = homozygous deletion, -1 = heterozygous deletion, 0 = normal copy, 1 = duplication, and 2 = amplification. For a gene pair (A, B), the co-loss event can be i) homCL: homozygous co-loss (-2, -2), ii) hetCL: heterozygous co-loss (-1, -1) or iii) mixCL: mixed co-loss (-2, -1 or -1, -2). For each co-loss event, we defined a fraction that quantifies the likelihood of the co-loss event. For instance, for the homozygous co-loss event, we defined the fraction for a gene pair A-B as f_1_ = n_homCL_/n_t_, where n_homCL_ is the number of patients with the homozygous co-loss of A-B and n_t_ is the total number of patients where A-B have a status as (-2, -2), (-2, 0) or (0,-2). We calculated the f_1_ of a gene pair without including samples that have homozygous deletions of more than 2000 genes (tail of the distribution in Figure A in S1 File). We noticed that several tumor samples have a very high number of homozygous deletions (Figure A in S1 File). Such samples can lead to an inflation of the co-loss likelihood regardless of whether they have an SL interaction or not. Similarly, we defined two fractions, f_2_ and f_3_, for heterozygous co-loss event and mixed co-loss events correspondingly (Table 1 and Fig 1). It should be noted that we did not use an approach in which we, in order to quantify under representation of co-loss events, compared the empirically observed co-loss rate of gene pair A-B with the product of the single loss rates for genes A and B. This approach assumes independency between the loss of randomly chosen genes, which is not what we observe (Figure B in S1 File).

**Table 1:** Five fractions derived from genomic variations for SL interaction identification.

f_1_ = n_homCL_/n_t_	$n_{homCL}=\{\begin{array}{c} A=-2\\ and\\ B=-2 \end{array}$	$n_{t}=\{\begin{array}{c} \begin{array}{c} A=-2,and\;B\;\in Z\\ or\\ A\;\in Z,and\;B=-2 \end{array} \end{array},\;where\;Z=0\;or-2$
f_2_ = n_hetCL_/n_t_	$n_{hetCL}=\{\begin{array}{c} A=-1\\ and\\ B=-1 \end{array}$	$n_{t}=\{\begin{array}{c} \begin{array}{c} A=-1,and\;B\;\in Z\\ or\\ A\;\in Z,and\;B=-1 \end{array} \end{array},\;where\;Z=0\;or-1$
f_3_ = n_mixCL_/n_t_	$n_{mixCL}=\{\begin{array}{c} \begin{array}{c} A=-2,and\;B=-1\\ or\\ A=-1,and\;B=-2 \end{array} \end{array}$	$n_{t}=\{\begin{array}{c} \begin{array}{c} A=-2,and\;B\;\in Z\\ or\\ A\;\in Z,and\;B=-2 \end{array} \end{array},\;where\;Z=0\;or-1$
f_4_ = n_co_under_/n_t_	$n_{co\_under}=\{\begin{array}{c} A=-1\\ and\\ B=-1 \end{array}$	$n_{t}=\{\begin{array}{c} \begin{array}{c} A=-1,and\;B\;\in Z\\ or\\ A\;\in Z,and\;B=-1 \end{array} \end{array},\;where\;Z=0\;or-1$
f_5_ = n_comp_/n_t_	$n_{comp}=\{\begin{array}{c} \begin{array}{c} A=-1,and\;B=1\\ or\\ A=1,and\;B=-1 \end{array} \end{array}$	$n_{t}=\{\begin{array}{c} \begin{array}{c} A=-1,and\;B\;\in Z\\ or\\ A\;\in Z,and\;B=-1 \end{array} \end{array},\;where\;Z=1,0\;or-1$

The variations in gene expression can be: -1 = under-expression, 0 = normal, and 1 = over-expression. Here, we defined two fractions, f_4_ and f_5_ (Table 1 and Fig 1). f_4_ quantifies the likelihood of both genes in a pair (A, B) are under-expressed. f_5_ is used to quantify how likely gene pair A-B has the expression up-down events, i.e., A is over expressed and B is under expressed or vice versa.

Here, each defined fraction is a signal where SL pairs show difference from non-SL pairs. For f_1_, f_2_, f_3_ and f_4_, we expected that SL pairs have smaller values for these fractions than non-SL pairs. However, for f_5_ we expected that SL pairs have larger values than non-SL pairs. To test these hypotheses, we compared the fractions in SL pairs with the fractions in non-SL pairs via one-sided Wilcoxon rank tests in R. We carried out four comparisons of homozygous deletion, heterozygous deletion, mixed deletion and co-underexpression to estimate the difference of co-loss tendency between SL and non-SL pairs. In the analysis of up-down compensation, we carried out two comparisons of expression up-down or genomic up-down. Bonferroni correction was used to correct for 4 multiple comparisons in the analysis of co-loss tendency and 2 multiple comparisons in the analysis of up-down compensation (p-values are indicated with P_adj._).

To validate the robustness of the signals, we compared the fractions in SL pairs to the fractions in random pairs. In each randomization, we first generated 300 random pairs from all human genes for which gene expression and CNV were available and then compared the mean of the fractions in the random pairs with the mean in SL pairs. We expected that the random pairs have a smaller mean of f_1_, f_2_, f_3_ or f_4_ but a larger mean of f_5_ than SL pairs. To test the hypotheses, we counted the randomizations (n_1_) where the difference of mean between the random pairs and SL pairs is contradictory to the expectation. For each comparison, we conducted 1000 randomizations and calculated the P-value for each hypothesis test as P = (n_1_+1)/1001.

### Under-sampling

The training set is significantly skewed with only 4.6% of the pairs belonging to the positive class (SL pairs) and the rest belonging to the negative class (non-SL pairs). Such a skewed training set can affect the performance of most standard classification algorithms[30]. Thus, we generated a more balanced training set by randomly under-sampling the negative class so that the number of gene pairs in it is equal to that of the positive class. The under-sampling is conducted with ROSE package in R[31] and repeated 100 times. All the classifiers in the study are trained on the balanced set.

### Construct the ensemble-based prediction model

We adopted an ensemble-based model to integrate the aforementioned 5 signals for predicting whether a gene pair has an SL interaction or not. The balanced training set (described above) was used to train the ensemble-based prediction model that combines multiple classifiers, namely AdaBoost, J48, LogitBoost, RandomForest, Logit, JRip and PART. The combination rule is simply based on the mean function, $p\left(x\right)=\frac{1}{N}\sum_{i=1}^{N}p_{i}\left(x\right)$, where x is a given gene pair and *p*_*i*_ *(x)* is the probability that x is predicted to be SL by classifier i. The probabilities *p*_*i*_ *(x)* from all classifiers, except for RandomForest, are obtained from the ‘RWeka’ package[32]. The RandomForest classifier is implemented with the ‘randomForest’ package in R[33].

To quantify the performance of the ensemble-based model, we used a 10-fold cross-validation framework on all empirically measured 270 SL pairs and 5660 non-SL pairs. In each cross-validation, the ensemble-based model is trained on nine of the randomly constructed 10 fractions and predictions are made for the test samples in the remaining fraction. The performance of the model in each cross-validation is evaluated by a ROC curve, the corresponding AUC score and a precision-recall curve. Repeating this procedure 10 times, a mean ROC curve, a mean AUC score and a mean precision-recall curve are calculated as the evaluation for the performance of the ensemble-based prediction model.

### Construction of the genome-wide human SL interaction map

To predict SL interactions in human at a genome-wide scale, we first selected 15620 genes that are measured for both CNV and mRNA variations in cancer cells. As mentioned in the results section, due to the presence of arm-level copy number variations, gene pairs on the same chromosome are more likely to be co-lost regardless of the status of SL interaction. Thus, we applied our model to ~115 million genes pairs that are located on different chromosomes. To construct a highly accurate SL interaction map, we predicted a list of more than 591,000 SL interactions based on a probability score (*p*(*x*)) threshold of 0.81, which achieved a precision of 67% at a recall of 10%.

## Results

### Synthetic lethal interactions are reflected in cancer genome evolution

We first asked whether empirically observed SL interactions are reflected in gene presence/absence and gene expression in cancer cells. To answer that, we used two types of genome variation from the Cancer Genome Atlas (TCGA)[22], i.e., i) copy number variations (CNVs) and ii) gene expression variations. The TCGA consortium measured 14136 tumor samples for CNVs and 7362 tumor samples for gene expression variations. To determine whether genes in cancer samples are significantly over- or under-expressed, we determined their expression-levels relative to normal samples of the same tissue type (Methods). We obtained the empirical SL interactions from two recent studies[23, 24] that measured SL interaction in colon tumor cell lines and have the highest genome coverage among all the studies available. In total we collected 270 SL pairs and 5660 non-SL pairs (S1 Table).

**Fig 2:**
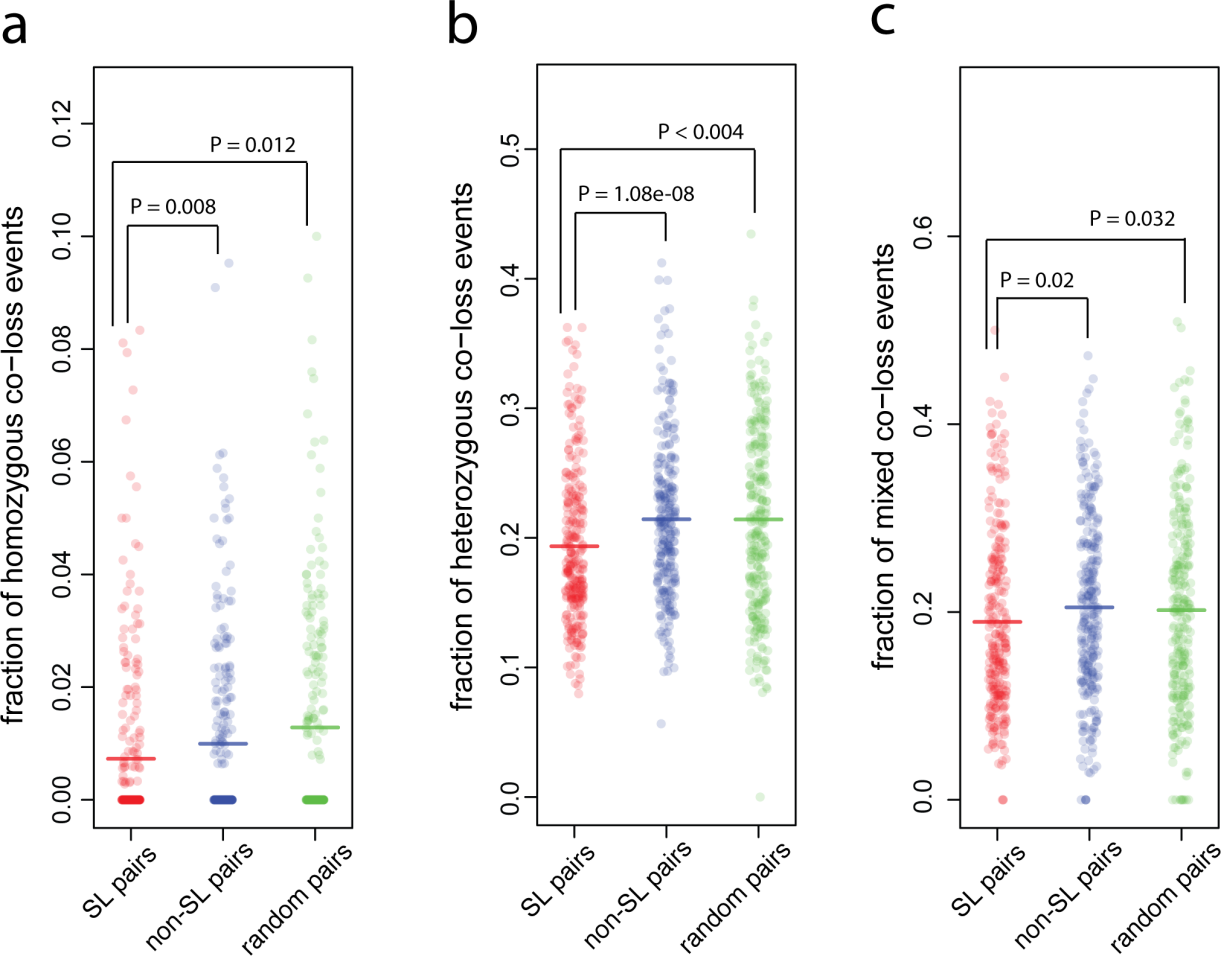
SL pairs are reflected in copy number variations. SL pairs are less likely to have **(a)** homozygous co-loss events, **(b)** heterozygous co-loss events and **(c)** mixed co-loss events than non-SL pairs or random pairs. The fractions for these three types of co-loss events are described as f_1_, f_2_, f_3_ in Methods and Fig 1. Each dot is the fraction for a given pair and the horizontal bar represents the mean of the fractions. P-values for the comparison between SL and non-SL pairs were calculated using one-sided Wilcoxon rank test. P-values for the comparison between SL and random pairs were calculated from 1000 randomizations. P-values were adjusted for multiple comparisons using the Bonferroni correction (see details in Methods).

We first tested whether SL pairs are less likely to be co-lost in a genome than non-SL pairs. A gene can either be homozygously or heterozygously deleted. We first focused on homozygous losses in which both copies of a gene are lost. We express the likelihood of homozygous co-loss of both genes in a gene pair by the fraction f = n_1_/n_2_, where n_1_ is the number of tumor samples with a co-loss of both genes and n_2_ is the number of tumor samples in which at least one gene is lost (see Methods and Fig 1). Indeed, we found that SL pairs are less likely to be homozygously co-lost than the non-SL pairs (0.00728 vs 0.0104, one-sided Wilcoxon rank test, P_adj._ = 0.008, Fig 2A).

We performed several additional analyses to show that this result is valid and robust. First, we showed that the difference in co-loss events is not caused by the difference in single gene loss rates. Indeed the homozygous gene deletion rate of the genes in SL pairs is not different from the deletion rate of the genes in non-SL pairs (0.00402 vs 0.00406, two-sided Wilcoxon rank test, P = 0.38). Secondly, given the limited genome coverage of the known SL and non-SL pairs available for our analysis, we also compared the likelihood of co-loss events of SL pairs with random pairs from the human genome. We found a significant difference in co-loss between SL pairs and random pairs (0.00728 vs 0.0128, 1000 randomizations, P_adj._ = 0.012, Fig 2A). This shows that the difference in the likelihood of co-loss events between the SL pairs and the random gene pairs is a consistent signal across the human genome. The difference between SL pairs and random pairs is larger than the difference between SL pairs and non-SL pairs (Fig 2A). This is likely due to the fact that the genes included in the experiments tend to be biased towards those that are frequently lost, i.e. the homozygous deletion rate of genes in SL/non-SL pairs is higher than that in random pairs (0.0049 vs 0.0042, one-sided Wilcoxon rank test, P = 0.04). It should furthermore be noted that we require the gene pairs included in the analysis to be composed of genes on different chromosomes. The reason for this is that the presence of arm-level copy number variations will always cause a high probability of co-loss for the gene pairs on the same chromosome regardless of whether they have an SL interaction or not.

Besides the homozygous co-loss, where both genes are homozygously deleted, there exist the possibilities of a heterozygous co-loss where both genes are heterozygously deleted and a mixed co-loss where one gene is homozygously deleted and the other is heterozygously deleted. For the heterozygous co-loss and for the mixed co-loss event we carried out the same analysis as done above for the homozygous co-losses. For both types of co-loss events, we found a significant and robust signal, i.e., the SL pairs are less likely to be co-lost than the non-SL pairs (for heterozygous co-loss 0.1935 vs 0.216, one-sided Wilcoxon rank test, P_adj._ = 1.08e-08, Fig 2B; for mixed co-loss 0.189 vs 0.2008, one-sided Wilcoxon rank test, P_adj._ = 0.02, Fig 2C). As was the case for the homozygous co-losses, both signals are consistent when SL pairs are compared with random gene pairs (for heterozygous co-loss 0.1925 vs 0.218, P_adj._ < 0.004, Fig 2B; for mixed co-loss 0.189 vs 0.210, P_adj._ = 0.032, Fig 2C).

**Fig 3:**
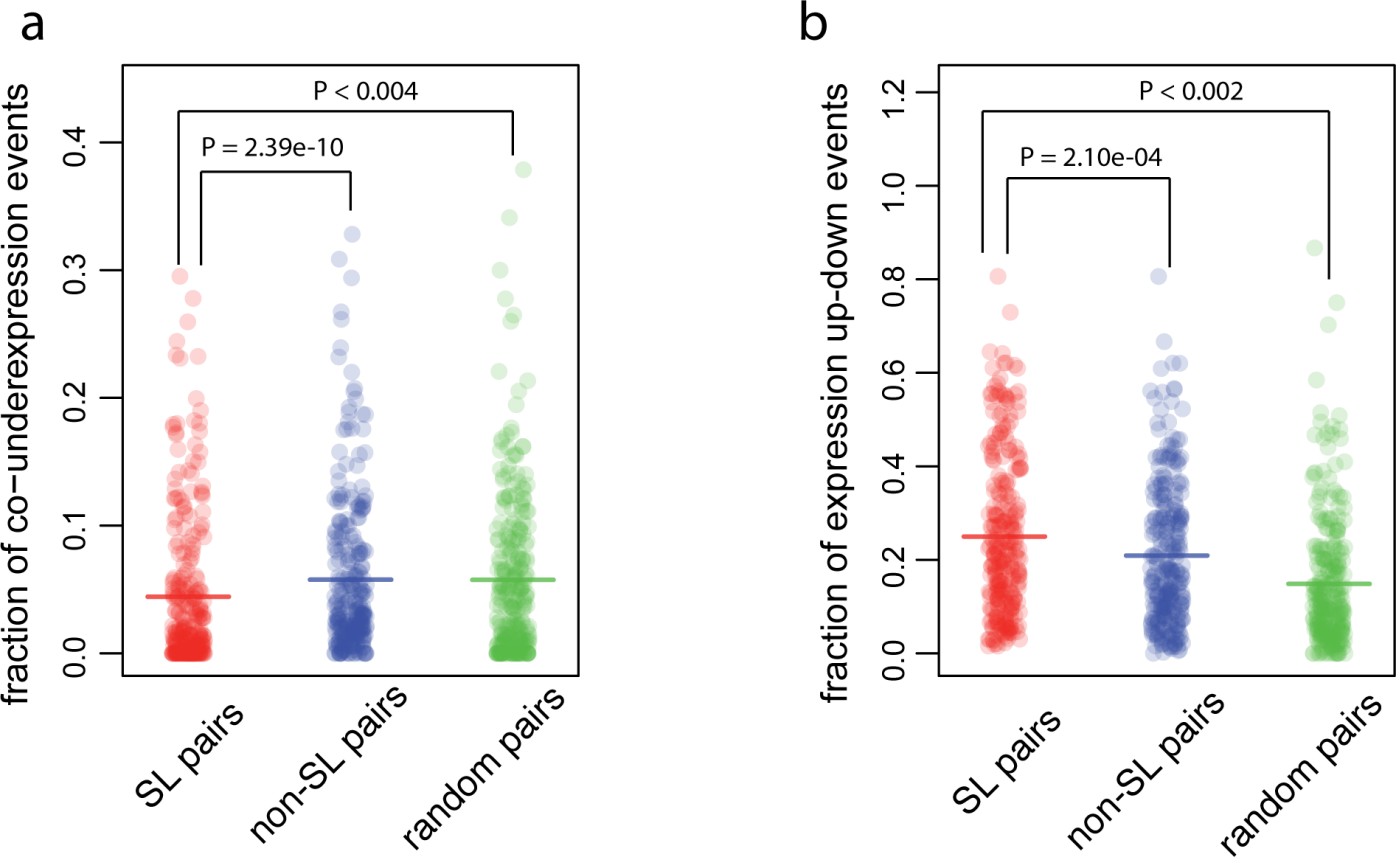
SL pairs are reflected in gene expression variations. **(a)** SL pairs are less likely to be co-underexpressed relative to the control i.e., non-SL or random pairs. The fraction for co-underexpression events is described as f_4_ in methods and Fig 1. **(b)** SL pairs are more likely to have expression up-down events where one gene is over-expressed while the other in under-expressed. The fraction for such pattern is described as f_5_ in Methods and Fig 1. Each dot is the fraction for a given pair and the horizontal bar represents the mean of the fractions. P-values for the comparison between SL and non-SL pairs were calculated with a one-sided Wilcoxon rank test. P-values for the comparison between SL and random pairs were calculated from 1000 randomizations. P-values were adjusted for multiple comparisons using the Bonferroni correction (for details see Methods).

We next examined gene expression levels, where we expected to find a similar signal to the one we found at the level of gene absence/presence, since the under-expression of one gene can also result in the loss of its activity. Indeed, we found that SL pairs are less likely to be both under-expressed than non-SL pairs (0.0443 vs 0.0586, one-sided Wilcoxon rank test, P_adj._ = 2.39e-10, Fig 3A). Only pairs composed of genes on different chromosomes are included in the analysis. Again the signal is consistent when SL pairs are compared with random gene pairs (0.0443 vs 0.0570, P_adj._ < 0.004, Fig 3A).

Previous studies [34, 35] have shown another pattern in genes in SL pairs at the transcription level. In this pattern one gene of an SL interacting pair is over-expressed while its partner is under-expressed. Thus, we expected that compared with non-SL pairs, SL pairs would have higher probabilities to have an expression pattern where one gene is over-expressed while the other is under-expressed. We refer to this as expression up-down. The probability of this expression pattern is quantified by the fraction f = n_1_/n_2_, where n_1_ is the number of tumor samples that have the pattern and the n_2_ is the number of tumor samples that have an under-expression of at least one of the genes (see Methods and Fig 1 for details). As expected, we found that SL pairs are more likely to have this expression pattern than non-SL pairs (0.250 vs 0.211, one-sided Wilcoxon rank test, P_adj._ = 2.10e-04, Fig 3B). Again, we validated the consistency of the signal by comparing the likelihood of this expression pattern in the SL pairs against its likelihood in random pairs (0.250 vs 0.146, 1000 randomizations, P_adj._ < 0.002, Fig 3B). We note that the difference between SL pairs and random pairs is higher than that between SL pairs and non-SL pairs. This is possibly due to the fact that the genes included in the experiments were biased towards those that are more likely to be over-expressed when one is mutated, i.e., the over-expression of genes in non-SL pairs is higher than that of random genes (0.0957 vs 0.0789, one-sided Wilcoxon rank test, P = 1.08e-06). We also analyzed a genomic pattern at the gene presence/absence level by calculating the probability for each gene pair to have a CNV pattern where one gene is duplicated or amplified while the other one is homozygously or heterozygously deleted, referred to as genomic up-down in the remainder of the text. We found that SL pairs indeed have a higher probability to have the genomic up-down combination at the DNA level than non-SL pairs (0.300 vs 0.274, one-sided Wilcoxon rank test, P_adj._ = 1.65e-07), but this is not significant when we compared the SL pairs to random gene pairs.

In total, we found five patterns in the CNVs and gene expression variations in cancer cells, all of which showed that synthetic lethal interactions are reflected in cancer genome evolution. These five patterns fall into two categories: i) genes in SL pairs are more likely to be over-expressed when their interaction partner is under-expressed and ii) genes in SL pairs are less likely to be co-lost either at the DNA level or at the gene expression level.

### An ensemble-based model for predicting synthetic lethal interactions

We next asked whether these five genomic patterns are strong enough to reliably predict SL pairs in human on a genome-wide scale. To do that we developed an ensemble-based model that integrates the five patterns. It should be noted that we did not include the genomic up-down pattern found in CNVs since SL pairs are not significantly different from random pairs. An ensemble-based model is a classifier that combines the prediction results from multiple classifiers, such as decision trees and logistic regression. It is known that such an ensemble-based model can improve performance relative to a single classification procedure[36], especially for complex problems such as SL prediction involving noisy inputs[37].

We used the empirically measured 270 SL pairs and 5660 non-SL pairs as described in the previous analysis. To construct the prediction model, we first needed to handle the imbalance of sample size between the negative class, i.e. non-SL pairs, and the positive class, i.e. SL pairs. The skewed distribution of the classes can affect the performance of prediction models [30]. To solve this issue, we randomly under-sampled the negative class (non-SL pairs, 95.4% of the training set) to produce a set of negative samples of the same size as the positive class (SL pairs, 4.6% of the training set). This balanced combination of two sets is used to train an ensemble-based model for SL prediction. Note that the under-sampling is only applied to the training set. In total we selected seven different single classifiers as the base for the ensemble model: AdaBoost[38], J48[39], LogitBoost[40], RandomForest[41], Logit[42], JRip[43] and PART[44] which are either robust against noisy data or over-fitting. After being trained with the balanced set, each single classifier generates a probability that a gene pair has an SL interaction. Then we integrated all seven probabilities from these single classifiers by calculating the mean of the seven probabilities and used that as the final predicted probability.

**Fig 4:**
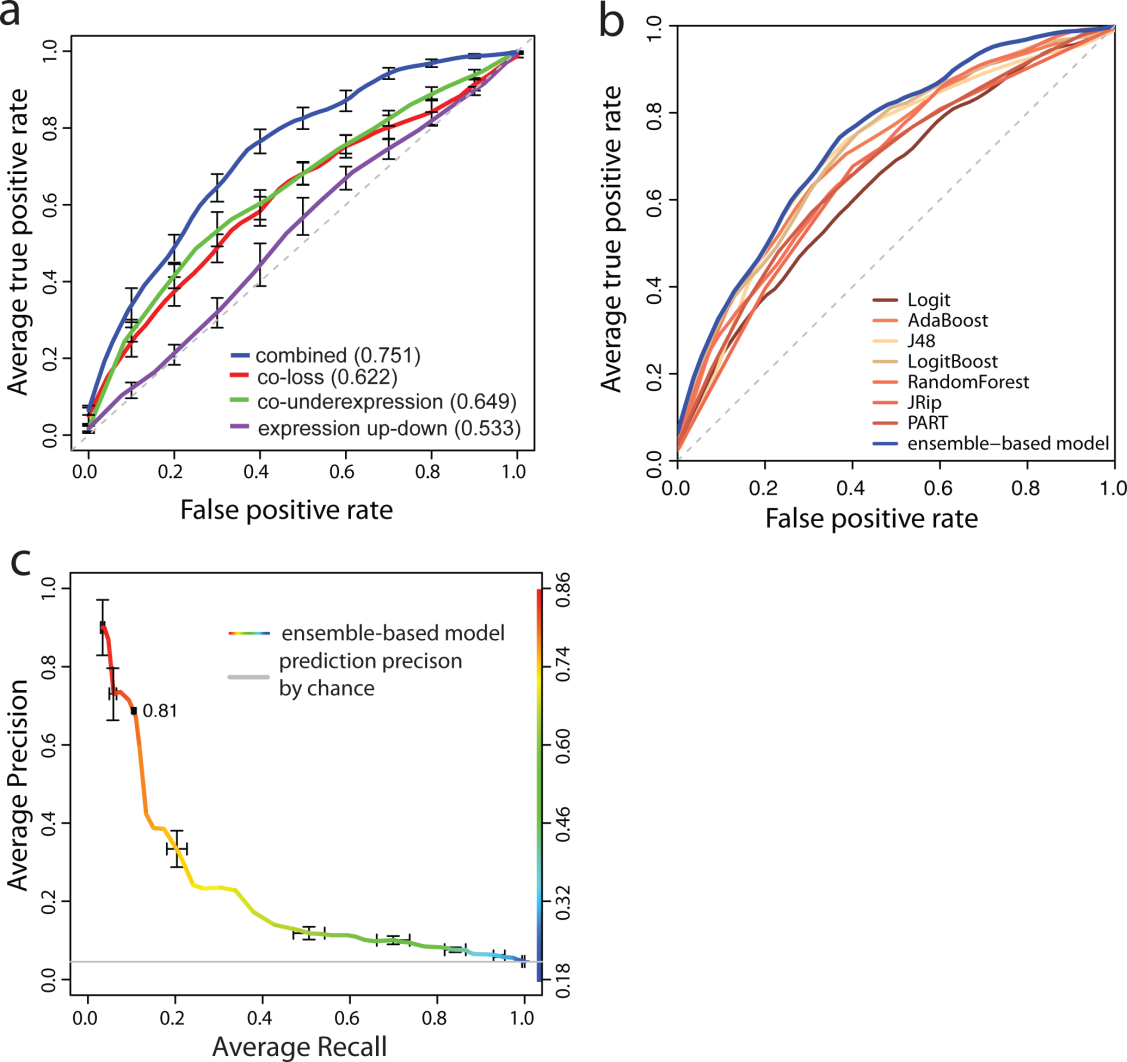
Receiver operating characteristic (ROC) curves. **(a)** The ensemble-based prediction model based on all five combined patterns has an area under curve (AUC) of 0.75 (blue line), which is estimated by 10-fold cross validation. Ensemble-based prediction models based on the non-combined individual patterns, i.e., co-loss in CNVs, co-underexpression and expression up-down, are shown in red, green and purple respectively, and have lower AUCs. Standard error bars are added to each ROC. **(b)** The ensemble-based prediction model (the blue ROC curve) has a better performance than all the seven single. **(c)** The precision and recall curve is estimated from 10-fold cross validation. Standard error bars are added. The curve is colored according to the cutoff of probability. The color panel of the probability is plotted at the right side. The cutoffs of probability scores (*p*(*x*)), 0.81, are printed at the corresponding curve positions. The grey line represents the prediction precision by chance alone.

To assess the performance of the ensemble-based prediction model, we used a 10-fold cross-validation on all the empirically measured 270 SL pairs and 5660 non-SL pairs. The plot of sensitivity (i.e., true positive rate) versus false positive rate of the ensemble-based model shows that our model achieves an area under ROC curve (AUC) of 0.75 (standard error = 0.016, Fig 4B). It should be noted that this high AUC is only achieved when combining all patterns (Fig 4A). We also found that the ensemble-based model achieved the highest AUC compared to all seven single classifiers (Fig 4B). In order to predict a genome-wide SL interaction map, we estimated the average precision and recall values from the 10-fold cross-validation (Fig 4C). We then applied the model to all gene pairs on the genome. Among ~115 million pairs for which gene expression and CNV data were available, we predicted more than 591,000 SL interactions based on a probability score threshold of 0.81 (Fig 4C), which corresponds to an estimated precision of 67% based on our training set, i.e., 14-fold higher than expected from chance (S1 Dataset). Note that the model achieves a similar precision (60% at p = 0.81) when using an independent set of experimentally measured SLs (Figure C in S1 File).

## Discussion

In this study we present a novel computational model that identifies SL interactions from cancer genomic data on a genome-wide scale. To develop such a model, we first systematically explored how SL interactions are reflected in cancer genomes and their gene expression levels. We found that compared with non-SL pairs, genes in SL pairs are significantly less likely to be co-lost in a cancer genome, both at the level of gene expression and at the level of copy number variation. Moreover, SL pairs are more likely to have an expression up-down pattern where one gene is over-expressed while the other one is under-expressed, which is consistent with another recent study[20]. Based on these results, we constructed an ensemble-based model to predict SL interactions via integrating these unique patterns in cancer genome variations, achieving a high prediction performance (AUC = 0.75). Our work presents a direct way to predict SL interactions from cancer genomic data, in contrast to most existing computational models, which identify SL interactions either specific for the model organisms yeast and *C*. *elegans* [16–18], or predict SL pairs in human in an indirect way by mapping SL interactions from yeast to human via orthology [45]. A strategy that uses human genomes by exploring the ‘compensation’ pattern also requires, as an additional criterion, that the genes are generally co-expressed [20]. As SLs have the characteristic that only one of the two genes is strictly needed, co-expression is not crucial. As such, co-expression as an additional criterion limits the coverage of SL interactions encoded in the genome, which is reflected in the total number of predicted SL pairs by Jerby-Arnon L *et al*. (2816 with accuracy of 0.779)[20]. In contrast, our approach, which does not rely on co-expression, predicts many more SL interactions with a comparable accuracy (591,000 with an estimated accuracy of 0.75).

Future work should focus on the following issues to improve the performance of the model. First, given the genomic and microenvironment heterogeneity among different types of tumors[46, 47], the empirically detected SL interactions included in our analysis might be only specific to colon cancer in which the experiments were carried out. As genetic interactions were found to be growth condition specific[48], it might be that two genes are co-lost in certain tumors simply because the functions of these SL pairs are not essential for that particular cancer type. Such discordance of tissue types might have dampened the effect size we discovered. To improve this, one can focus on gene-expression and CNVs that are taken from the same tumor type as the empirical SLs. A model can then be constructed to predict tumor type specific SLs, which is valuable to overcome the challenges posed by inter-tumor heterogeneity in cancer treatments. Secondly, our model only considered gain or loss of gene function caused by CNVs and variations in gene expression. There are other mutations that can result in gain or loss of gene functions, such as mutations of miRNA[49, 50] and epigenetic mutations[51, 52]. When knowledge becomes available on how these other types of genomic variations affect gene function and genetic interactions, these mutations could also be taken into account. Thirdly, our model achieves a good prediction performance by a 10-fold cross validation. However, we note that the model is trained on a relative small number of available SL and nonSL pairs, which constraints a precise estimation of the model performance for genome-wide prediction. The performance can be better estimated in the future when more empirically measured SLs become available. Finally, it still remains to be seen to what extent these predicted SL interactions from cancer genomes are relevant to understand other diseases. For diseases where CNV or gene expression data are available, one can prioritize disease-associated SL interactions from our prediction list by selecting pairs that are co-lost in the disease.

Taken together, we systematically investigated and showed that SL interactions are reflected in genome evolution of cancer in various forms. Based on the unique patterns discovered in cancer genomics, we proposed a simple approach to identify SL, which strongly improves existing frameworks. We generated a unique SL interaction network in human at the genome-scale covering up to 591,000 pairs with a high estimated precision. In the light of medical genetics, this list is highly valuable in the search for anti-cancer drug targets and in understanding human diseases.

## Supporting Information

S1 Dataset The list of predicted synthetic lethal interactions at prediction precision 0.67. (TXT)

S1 File Supplementary figures A, B, C. (DOCX)

S1 Table Neg noNeg list. (XLSX)

S2 Table The number of normal samples of different tissue types in RNAseq data. (XLSX)

## References

[1] Hartman JL, Garvik B, Hartwell L. Principles for the Buffering of Genetic Variation. Science. 2001;291(5506):1001–4. 10.1126/science.1056072 11232561

[2] Hermisson J, Wagner GnP. The Population Genetic Theory of Hidden Variation and Genetic Robustness. Genetics. 2004;168(4):2271–84. 10.1534/genetics.104.029173 15611191 PMC1448756

[3] Zuk O, Hechter E, Sunyaev SR, Lander ES. The mystery of missing heritability: Genetic interactions create phantom heritability. Proc Natl Acad Sci U S A. 2012. 10.1073/pnas.1119675109 PMC3268279 22223662

[4] Bloom JS, Ehrenreich IM, Loo WT, Lite T-LV, Kruglyak L. Finding the sources of missing heritability in a yeast cross. Nature. 2013;494(7436):234–7. 10.1038/nature11867 23376951 PMC4001867

[5] Hemani G, Shakhbazov K, Westra H-J, Esko T, Henders AK, McRae AF, et al Detection and replication of epistasis influencing transcription in humans. Nature. 2014;508(7495):249–53. 10.1038/nature13005 24572353 PMC3984375

[6] Fedeles SV, Tian X, Gallagher A-R, Mitobe M, Nishio S, Lee SH, et al A genetic interaction network of five genes for human polycystic kidney and liver diseases defines polycystin-1 as the central determinant of cyst formation. Nat Genet. 2011;43(7):639–47. 10.1038/ng.860 21685914 PMC3547075

[7] Nakatomi M, Wang X-P, Key D, Lund JJ, Turbe-Doan A, Kist R, et al Genetic interactions between Pax9 and Msx1 regulate lip development and several stages of tooth morphogenesis. Dev Biol. 2010;340(2):438–49. 10.1016/j.ydbio.2010.01.031 20123092

[8] Bryant HE, Schultz N, Thomas HD, Parker KM, Flower D, Lopez E, et al Specific killing of BRCA2-deficient tumours with inhibitors of poly(ADP-ribose) polymerase. Nature. 2005;434(7035):913–7. 15829966 10.1038/nature03443

[9] Ashworth A, Lord CJ, Reis-Filho JS. Genetic Interactions in Cancer Progression and Treatment. Cell. 2011;145(1):30–8. 10.1016/j.cell.2011.03.020 21458666

[10] Kapoor A, Yao W, Ying H, Hua S, Liewen A, Wang Q, et al Yap1 Activation Enables Bypass of Oncogenic Kras Addiction in Pancreatic Cancer. Cell. 2014;158(1):185–97. 10.1016/j.cell.2014.06.003 24954535 PMC4109295

[11] McManus KJ, Barrett IJ, Nouhi Y, Hieter P. Specific synthetic lethal killing of RAD54B-deficient human colorectal cancer cells by FEN1 silencing. Proc Natl Acad Sci U S A. 2009;106(9):3276–81. 10.1073/pnas.0813414106 19218431 PMC2651317

[12] Roguev A, Talbot D, Negri GL, Shales M, Cagney G, Bandyopadhyay S, et al Quantitative genetic-interaction mapping in mammalian cells. Nat Meth. 2013;10(5):432–7. 10.1038/nmeth.2398 PMC3641890 23407553

[13] Horn T, Sandmann T, Fischer B, Axelsson E, Huber W, Boutros M. Mapping of signaling networks through synthetic genetic interaction analysis by RNAi. Nat Meth. 2011;8(4):341–6. 10.1038/nmeth.1581 21378980

[14] Costanzo M, Baryshnikova A, Bellay J, Kim Y, Spear ED, Sevier CS, et al The Genetic Landscape of a Cell. Science. 2010;327(5964):425–31. 10.1126/science.1180823 20093466 PMC5600254

[15] Babu M, Arnold R, Bundalovic-Torma C, Gagarinova A, Wong KS, Kumar A, et al Quantitative Genome-Wide Genetic Interaction Screens Reveal Global Epistatic Relationships of Protein Complexes in *Escherichia coli* . PLoS Genet. 2014;10(2):e1004120 10.1371/journal.pgen.1004120 24586182 PMC3930520

[16] Zhong W, Sternberg PW. Genome-Wide Prediction of C. elegans Genetic Interactions. Science. 2006;311(5766):1481–4. 10.1126/science.1123287 16527984

[17] Wong SL, Zhang LV, Tong AHY, Li Z, Goldberg DS, King OD, et al Combining biological networks to predict genetic interactions. Proc Natl Acad Sci U S A. 2004;101(44):15682–7. 10.1073/pnas.0406614101 15496468 PMC524818

[18] Pandey G, Zhang B, Chang AN, Myers CL, Zhu J, Kumar V, et al An Integrative Multi-Network and Multi-Classifier Approach to Predict Genetic Interactions. PLoS Comput Biol. 2010;6(9):e1000928 10.1371/journal.pcbi.1000928 20838583 PMC2936518

[19] Dixon SJ, Fedyshyn Y, Koh JLY, Prasad TSK, Chahwan C, Chua G, et al Significant conservation of synthetic lethal genetic interaction networks between distantly related eukaryotes. Proc Natl Acad Sci U S A. 2008;105(43):16653–8. 10.1073/pnas.0806261105 18931302 PMC2575475

[20] Jerby-Arnon L, Pfetzer N, Waldman YY, McGarry L, James D, Shanks E, et al Predicting Cancer-Specific Vulnerability via Data-Driven Detection of Synthetic Lethality. Cell. 2014;158(5):1199–209. 10.1016/j.cell.2014.07.027 25171417

[21] Lu X, Kensche PR, Huynen MA, Notebaart RA. Genome evolution predicts genetic interactions in protein complexes and reveals cancer drug targets. Nat Commun. 2013;4 10.1038/ncomms3124 PMC3717498 23851603

[22] The results shown here are in whole or part based upon data generated by the TCGA Research Network: http://cancergenome.nih.gov/.

[23] Laufer C, Fischer B, Billmann M, Huber W, Boutros M. Mapping genetic interactions in human cancer cells with RNAi and multiparametric phenotyping. Nat Meth. 2013;10(5):427–31. 10.1038/nmeth.2436 23563794

[24] Vizeacoumar FJ, Arnold R, Vizeacoumar FS, Chandrashekhar M, Buzina A, Young JTF, et al A negative genetic interaction map in isogenic cancer cell lines reveals cancer cell vulnerabilities. Mol Syst Biol. 2013;9(1). 10.1038/msb.2013.54 PMC3817404 24104479

[25] Gao J, Aksoy BA, Dogrusoz U, Dresdner G, Gross B, Sumer SO, et al Integrative Analysis of Complex Cancer Genomics and Clinical Profiles Using the cBioPortal. Sci Signal. 2013;6(269):pl1-pl. 10.1126/scisignal.2004088 23550210 PMC4160307

[26] Broad Institute TCGA Genome Data Analysis Center (2014): Analysis Overview for 16 March 2014. Broad Institute of MIT and Harvard.

[27] Li B, Dewey C. RSEM: accurate transcript quantification from RNA-Seq data with or without a reference genome. BMC Bioinformatics. 2011;12(1):323 PubMed 10.1186/1471-2105-12-323 21816040 PMC3163565

[28] Benjamini Y, Hochberg Y. Controlling the False Discovery Rate: A Practical and Powerful Approach to Multiple Testing. J R Statist Soc B. 1995;57(1):289–300. 10.2307/2346101

[29] Benjamini Y, Yekutieli D. The control of the false discovery rate in multiple testing under dependency. The Annals of Statistics. 2001;29(4):1165–88. 10.1214/aos/1013699998

[30] Menardi G, Torelli N. Training and assessing classification rules with imbalanced data. Data Min Knowl Disc. 2014;28(1):92–122. 10.1007/s10618-012-0295-5

[31] Torelli NLaGMaN. a Package for Binary Imbalanced Learning. R Journal. 2014;6(1):82–92.

[32] Hornik K, Buchta C, Zeileis A. Open-source machine learning: R meets Weka. Comput Stat. 2009;24(2):225–32. 10.1007/s00180-008-0119-7

[33] Wiener ALaM. Classification and Regression by randomForest. R News. 2002;2(3):18–22.

[34] DeLuna A, Springer M, Kirschner MW, Kishony R. Need-Based Up-Regulation of Protein Levels in Response to Deletion of Their Duplicate Genes. PLoS Biol. 2010;8(3):e1000347 10.1371/journal.pbio.1000347 20361019 PMC2846854

[35] Kafri R, Bar-Even A, Pilpel Y. Transcription control reprogramming in genetic backup circuits. Nat Genet. 2005;37(3):295–9. 15723064 10.1038/ng1523

[36] Rokach L. Ensemble-based classifiers. Artif Intell Rev. 2010;33(1–2):1–39. 10.1007/s10462-009-9124-7

[37] Dietterich T. Ensemble Methods in Machine Learning. Multiple Classifier Systems Lecture Notes in Computer Science. 1857: Springer Berlin Heidelberg; 2000 p. 1–15.

[38] Freund Y, Schapire RE. A Decision-Theoretic Generalization of On-Line Learning and an Application to Boosting. Journal of Computer and System Sciences. 1997;55(1):119–39. 10.1006/jcss.1997.1504

[39] Quinlan JR. C4.5: Programs for Machine Learning: Morgan Kaufmann Publishers Inc; 1993. 302 p.

[40] Friedman J, Hastie T, Tibshirani R. Additive logistic regression: a statistical view of boosting. The Annals of Statistics. 2000;28(2):337–407. 10.1214/aos/1016218223

[41] Breiman L. Random Forests. Mach Learn. 2001;45(1):5–32. 10.1023/a:1010933404324

[42] Cessie L, van Houwelingen JC. Ridge Estimators in Logistic Regression. Appl Statist. 1992;41(1):191–201. doi: citeulike-article-id:4975518.

[43] Cohen WW. Fast Effective Rule Induction. In Proceedings of the Twelfth International Conference on Machine Learning: Morgan Kaufmann; 1995. p. 115–123.

[44] Frank E, Witten IH. Generating Accurate Rule Sets Without Global Optimization. Proceedings of the Fifteenth International Conference on Machine Learning. 657305: Morgan Kaufmann Publishers Inc.; 1998. p. 144–51.

[45] Deshpande R, Asiedu MK, Klebig M, Sutor S, Kuzmin E, Nelson J, et al A Comparative Genomic Approach for Identifying Synthetic Lethal Interactions in Human Cancer. Cancer Res. 2013;73(20):6128–36. 10.1158/0008-5472.can-12-3956 23980094 PMC3809957

[46] Burrell RA, McGranahan N, Bartek J, Swanton C. The causes and consequences of genetic heterogeneity in cancer evolution. Nature. 2013;501(7467):338–45. 10.1038/nature12625 24048066

[47] Junttila MR, de Sauvage FJ. Influence of tumour micro-environment heterogeneity on therapeutic response. Nature. 2013;501(7467):346–54. 10.1038/nature12626 24048067

[48] Harrison R, Papp B, Pal C, Oliver SG, Delneri D. Plasticity of genetic interactions in metabolic networks of yeast. Proc Natl Acad Sci U S A. 2007;104(7):2307–12. 10.1073/pnas.0607153104 17284612 PMC1892960

[49] Lu J, Clark AG. Impact of microRNA regulation on variation in human gene expression. Genome Res. 2012. 10.1101/gr.132514.111 PMC3396366 22456605

[50] Pasquinelli AE. MicroRNAs and their targets: recognition, regulation and an emerging reciprocal relationship. Nat Rev Genet. 2012;13(4):271–82. 10.1038/nrg3162 22411466

[51] Jaenisch R, Bird A. Epigenetic regulation of gene expression: how the genome integrates intrinsic and environmental signals. Nat Genet. 2003;33(3s):245–54. 10.1038/ng1089 12610534

[52] Herman JG, Baylin SB. Gene Silencing in Cancer in Association with Promoter Hypermethylation. N Engl J Med. 2003;349(21):2042–54. 10.1056/NEJMra023075 14627790

