# Supplementary material for: Predicting Human Genetic Interactions from Cancer Genome Evolution
Source: PLoS One. 2015 May 1;10(5):e0125795. doi: 10.1371/journal.pone.0125795 (PMC4416779; doi:10.1371/journal.pone.0125795)
Supplement: S1 File — (DOCX) [file pone.0125795.s002.docx]

**Figure A in S1 File: Distribution of the number of homozygous deletions in each tumor sample.**


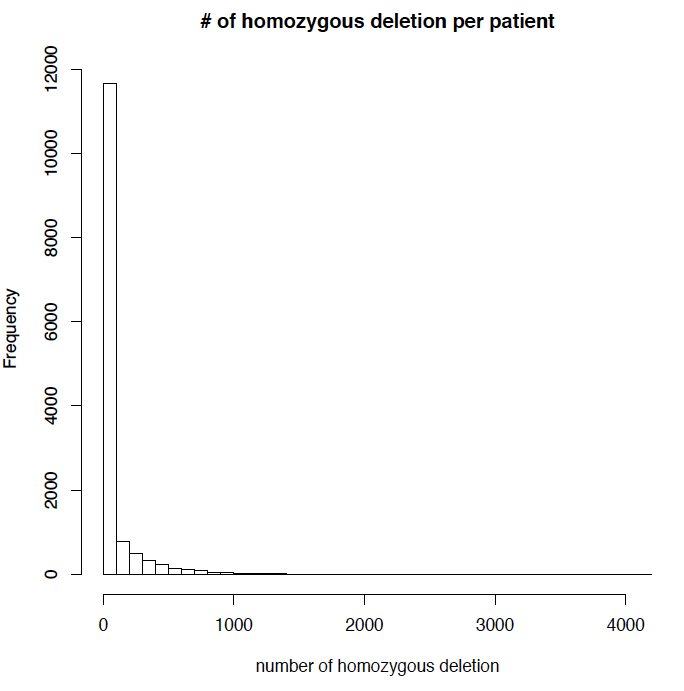


**Figure B in S1 File: Difference of expected and observed double mutation rate in random pairs.**


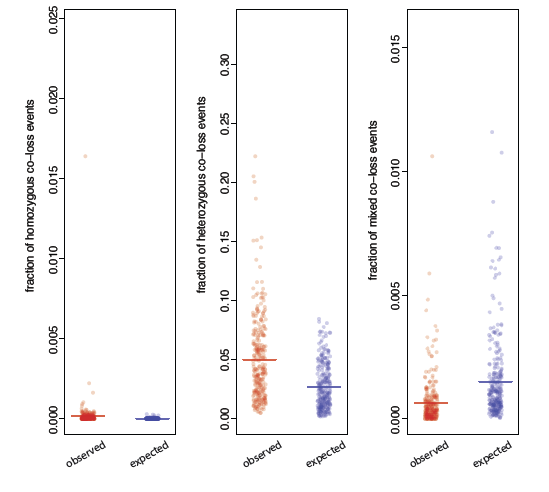


The observed fractions of the co-loss events are different from the expected fractions for random gene pairs. Upon the assumption of independency between random gene pair A-B, the expected fraction of co-loss events are calculated as the product of the single loss rates for gene A and B.

**Figure C in S1 File: Precision-recall plot.**


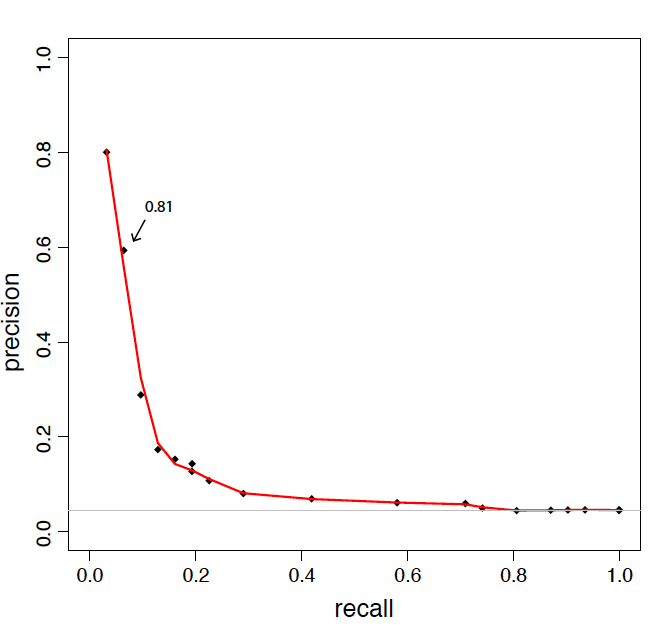


Precision estimation based on experimentally determined SLs from Pubmed_ID: 18388863, 15829967, 18832051, 18948595, 20049736, 24954535, 21642980, 19218431, 24556366, 11600719, 24568598, 23620515, 22895339
